# Supplementary material for: Antiproliferative activity of Grewia villosa ethyl acetate extract on cervical cancer HeLa cell line: Mechanistic insights through network pharmacology and functional assays approach
Source: PLoS One. 2025 Sep 24;20(9):e0331649. doi: 10.1371/journal.pone.0331649 (PMC12459836; doi:10.1371/journal.pone.0331649)
Supplement: S1 File — We used doxorubicin hydrochloride as an experimental positive control. Doxorubicin hydrochloride was tested on Vero-ccl-81 cells at different concentrations to enable estimation of CC50. NC: represent negative control (0.4% DMSO). S2 Fig. Anti-proliferative effects of the positive control. We used doxorubicin hydrochloride as an experimental positive control. Doxorubicin hydrochloride was tested on cancerous HeLa cells at different concentrations to enable estimation of IC50. NC: represent negative control (0.4% DMSO). (DOCX) [file pone.0331649.s001.docx]

**Antiproliferative activity of *Grewia villosa* ethyl acetate extract on cervical cancer HeLa cell line: Mechanistic insights through network pharmacology and functional assays approach**

Sally Wambui Kamau^1,2*^, Mercy Jepkorir^2^, Gilbert Kipkoech^1,2^, Inyani John Lino Lagu^4^, Wesley Kanda^2^, Susan Kibunja^1,2^, Rakita Letoluo^2^, Shadrack Barmasai^3^, Alice Wanyoko^2^, Vincent Ruttoh^3^, James Kuria^2^, Peter Githaiga Mwitari^2^, Mathew Piero Ngugi^1^ and Sospeter Ngoci Njeru^2,5*^

^1^Department of Biochemistry, Microbiology and Biotechnology, Kenyatta

University, Nairobi, Kenya.

^2^Centre for Traditional Medicine and Drug Research (CTMDR), Kenya Medical Research Institute (KEMRI), Nairobi, Kenya.

^3^Centre for Virus Research (CVR), Kenya Medical Research Institute (KEMRI), Nairobi, Kenya.

^4^Pan African Union Institute for Basic Science and Technology (PAUSTI), Nairobi, Kenya.

^5^Centre for Community Driven Research (CCDR), Kenya Medical Research Institute (KEMRI), Kirinyaga, Kenya.

*Co-corresponding authors

*snjeru@kemri.go.ke and [salliekamau@gmail.com](mailto:salliekamau@gmail.com)


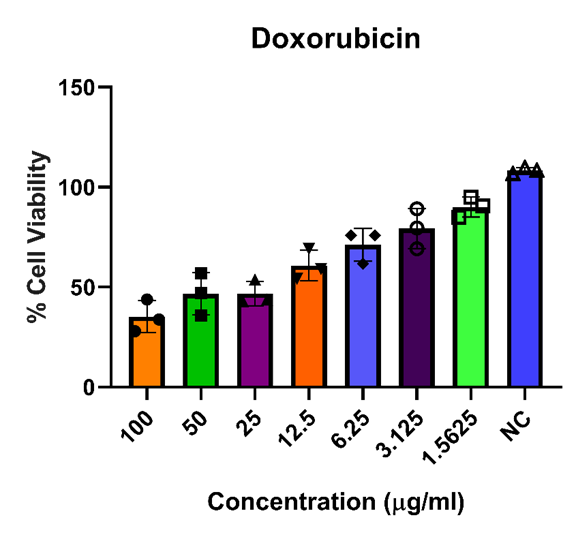


**S1 Fig.** **Cytotoxic effects of the experimental positive control**. We used doxorubicin hydrochloride as an experimental positive control. Doxorubicin hydrochloride was tested on Vero-ccl-81 cells at different concentrations to enable estimation of CC_50_. NC: represent negative control (0.4% DMSO)


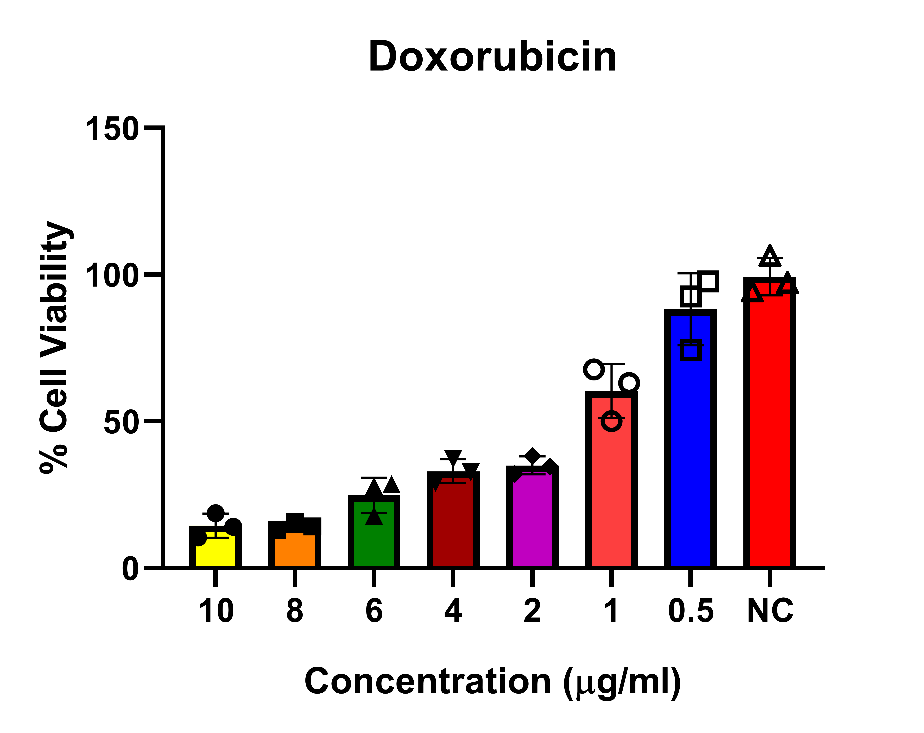


**S2 Fig.** **Anti-proliferative effects of the positive control**. We used doxorubicin hydrochloride as an experimental positive control. Doxorubicin hydrochloride was tested on cancerous HeLa cells at different concentrations to enable estimation of IC_50_. NC: represent negative control (0.4% DMSO)
