# Supplementary material for: Antiproliferative activity of Grewia villosa ethyl acetate extract on cervical cancer HeLa cell line: Mechanistic insights through network pharmacology and functional assays approach
Source: PLoS One. 2025 Sep 24;20(9):e0331649. doi: 10.1371/journal.pone.0331649 (PMC12459836; doi:10.1371/journal.pone.0331649)
Supplement: Attachment — Submitted filename: 2.1 RESPONSE TO REVIEWERS.docx [file pone.0331649.s004.docx]

**Response to Editor and Reviewers:**

Thank you for taking the time to review our manuscript and make additional suggestions. We have taken your comments seriously and addressed them as shown in the table below.

Your comments have helped us to improve this manuscript substantially, and are persuaded it is now ripe for publication. Thank you for your continued support.

Sincerely’

Sospeter (for the co-authors)

**EDITORS COMMENTS**

| **COMMENTS** | **RESPONSES/REBUTTAL** |
| --- | --- |
| Thank you for submitting your manuscript to PLOS ONE. After careful consideration, we feel that it has merit but does not fully meet PLOS ONE’s publication criteria as it currently stands. Therefore, we invite you to submit a revised version of the manuscript that addresses the points raised during the review process | Thank you, we have addressed the concerns raised by the reviewer. |
| Please include the following items when submitting your revised manuscript:   - A rebuttal letter that responds to each point raised by the academic editor and reviewer(s). You should upload this letter as a separate file labeled 'Response to Reviewers'. - A marked-up copy of your manuscript that highlights changes made to the original version. You should upload this as a separate file labeled 'Revised Manuscript with Track Changes'. - An unmarked version of your revised paper without tracked changes. You should upload this as a separate file labeled 'Manuscript'. | This is done  This is done  This is done |
| If the reviewer comments include a recommendation to cite specific previously published works, please review and evaluate these publications to determine whether they are relevant and should be cited. There is no requirement to cite these works unless the editor has indicated otherwise. | NA |
| Please review your reference list to ensure that it is complete and correct. If you have cited papers that have been retracted, please include the rationale for doing so in the manuscript text, or remove these references and replace them with relevant current references. Any changes to the reference list should be mentioned in the rebuttal letter that accompanies your revised manuscript. If you need to cite a retracted article, indicate the article’s retracted status in the References list and also include a citation and full reference for the retraction notice. | References checked and are all fine |
| The reviewer raised some minor questions that need to be addressed. | I have addressed them all as shown below |

**COMMENTS FROM REVEIWER #1**

| **COMMENTS** | **RESPONSES** |
| --- | --- |
| Abstract  Authors were indicated that this study is a first study which reported antiprolifirated activity of Grewia villosa in cervical cancer. It is recommended to write that knowledge is limited or absent. It is impossible to be sure that the research is unique. | This was captured in the tracked copy before corrections were made, but was already rectified in the clean copy. I have highlighted (from line 45 and 87) how it reads, and we have avoided the mention of being first study throughout the document.  On line 87, I have modified the sentence to read “Given the lack of scientific data… ” |
| Introduction  Chemotherapy is not primary treatment of cervical cancer. Chemotherapy alone recommended as a primary treatment in advanced stage of cervical cancer without PDl mutation | We have corrected this and appended a new different reference and now reads “Chemotherapy is usually used as an integral part of the standard cervical cancer treatment regimen, either as an adjuvant therapy following surgery to forestall recurrence, in combination with radiotherapy, or as a standalone therapy for locally advanced cervical cancer without PDl mutation [10]”…line 68 |
| Methods  Chapter methods clearly written  Line 190 Recommended to clarify of photochemical screening test of GVEA  There is no presented information on Ethics | We have added more information to make this section more clear, and now reads “The qualitative phytochemical screening tests of GVEA extract were conducted, and classes of phytochemicals were identified through characteristic color changes according to standard methods. Particularly, sulfuric acid solution and Mayer’s reagent test were used to identify alkaloids, water frothing and olive oil emulsion test were used to identify saponins, alkaline reagent (aqueous ammonia solution) test was used to identify flavonoids, Salkowski test was used to identify terpenoids, Keller-Killiani test was used to identify glycosides, ferric chloride and lead acetate test were used to identify tannins, and sodium hydroxide and ferric chloride tests were used to identify phenolics [28-30].”  According to the Plos One Journal instructions, the information on ethics is usually captured in the online submission system and not on the attached document. We have already captured the ethics statement in the online submission system, and it reads as “Approval for this study was granted by the Scientific Ethical Review Unit of the Kenya Medical Research Institute (KEMRI/SERU/CTMDR/104/4466)” |
| Results  Data performed chronologically  Data on chapter and in figures/tables equal  There is an interpretation of the results in all sub chapters  Line 284 Why authors decided to examine doxorubicine in control group. Why cisplatin is not used for control group as a chemotherapy drug choice according to guidelines | Thank you for compliment  The reviewer is correct that Cisplatin is a widely used chemotherapeutic drug for cervical cancer; however, the literature also indicates that cisplatin and doxorubicin are the two most commonly used drugs (<https://dx.doi.org/10.13005/bpj/3133>). This publication has recently demonstrated that doxorubicin exhibits higher activity and selectivity than cisplatin, indicating that it provides a more suitable comparator. Secondly, doxorubicin was the control available in our labs. We will capture this aspect as a limitation of the study, see line 656 “4) We also used doxorubicin as our positive control, even though cisplatin is the widely used chemotherapeutic agent for the treatment of advanced cervical cancer. In the future, it is recommended to consider using cisplatin as an alternative to or in addition to doxorubicin as a comparator. Notably, both chemotherapeutic agents are used in the treatment of advanced cervical cancer, and a recent study has demonstrated that doxorubicin is more active on HeLa cells and more selective on Vero cells than cisplatin [84], suggesting its possible superiority as a comparator on these cell line models.”. |
| Discussion  1. Chapter is not started from concise statement summarizing the main findings of the study  2. Strengths and weaknesses of study are presented clearly in chapter  3. Highlighted statement what is offered by the study for future direction | We chose a different approach of starting the section by laying the foundation, giving the finding and ending the section with the concise summary of the main finding. This is considering we have offered the same at the end of abstract, introduction and as part of our conclusing.  Thank you for positive affirmation  Thank you for positive affirmation |
